# Smart Tactical Application Tourniquet Versus Combat Application Tourniquet: Comparing Layperson Applications for Arterial Occlusion After a Video Demonstration

**DOI:** 10.7759/cureus.42615

**Published:** 2023-07-28

**Authors:** Robert L Gabbitas, Brandon M Carius

**Affiliations:** 1 Emergency Medicine, Irwin Army Community Hospital, Fort Riley, USA; 2 Emergency Medicine, Madigan Army Medical Center, Fort Lewis, USA

**Keywords:** prehospital trauma, trauma, bleeding control, hemorrhage, education

## Abstract

Background: Tourniquet efficacy for extremity hemorrhage is well established, although literature demonstrates variable first responder application efficacy. Several newer models, including the Smart Tactical Application Tourniquet (STAT, STAT Medical Devices, LLC, Freehold, New Jersey), offer alternatives to well-established devices like the Combat Application Tourniquet (CAT, C-A-T Resources, LLC, Rock Hill, South Carolina). Newer models are commercially sold without significant literature regarding efficacy or user feedback. We developed a pilot study to compare CAT and STAT applications for layperson hemorrhage control efficacy after a brief, standardized instructional video.

Study design and methods: This is a prospective randomized observational study that utilized layperson volunteers for the application of STAT or CAT. After a demographic survey, volunteers were randomized and watched the respective tourniquet instructional video, then applied the tourniquet to a HapMed hemorrhage simulator. The application was assessed for trial time, time to hemorrhage control, occlusion pressure, and total blood loss. Investigators also evaluated volunteers for proper application and received user feedback.

Results: Eighty-four total volunteers (42 CAT, 42 STAT) completed testing. Volunteers applied the CAT (50.0%, n = 21) with significantly greater rates of success than the STAT (0%, n = 0, *p *< 0.001). The CAT demonstrated significantly greater average occlusion pressure compared to the STAT (409.9 mm Hg vs. 116.5 mm Hg, *p* < 0.001). Similarly, CAT application resulted in significantly less average blood loss compared to the STAT (577.8 mL vs. 974.6 mL, *p* < 0.001). On the 5-point Likert scale, volunteers reported significantly higher benefits from video instruction and comfort with tourniquet application with the CAT over the STAT (4.7 vs. 4.0, *p* < 0.001, 4.0 vs. 2.4, *p* < 0.001).

Conclusions: When performed by laypersons with minimal video instruction, the CAT was applied with significantly higher rates of success, higher mean occlusion pressures, reduced blood loss, and higher end-user ratings than the STAT.

## Introduction

Tourniquets demonstrate effective management of extremity hemorrhage both on military battlefields and in civilian trauma [1-6]. The continued evolution of these devices aims to match effective mechanical leverage with user intuition and efficient application. While battlefield tourniquet employment remains high in the prehospital setting, recent data illustrate inadequate use and poor skill retention by civilian laypersons [7,8]. Although the Department of Homeland Security instituted the first "Stop the Bleed" campaign in 2015 to provide domestic and international bystander education on hemorrhage control, including tourniquets, success rates remain as low as 17% [9-11].

The Committee on Tactical Combat Casualty Care (CoTCCC) recommends multiple commercial tourniquets for extremity hemorrhage control [12]. This includes the Combat Application Tourniquet, more often referred to as CAT (C-A-T Resources, LLC, Rock Hill, South Carolina), one of the original modern tourniquets and often used as a reference for newer novel tourniquet devices [13-16]. However, poor user intuition, a lack of education retention, and a confidence-competence mismatch result in improper CAT application, hampering its proven efficacy for arterial occlusion [7-9,17]. Nevertheless, the CAT is still largely the standard in civilian prehospital hemorrhage control and is featured as the primary tourniquet device for the Stop the Bleed Course, including being featured for sale on the organization's website.

A burgeoning market for prehospital medical care motivates continuous innovation and evolution of materials and device mechanics, and many are sold without formal evaluation or review by authoritative bodies [18]. This growing list of devices purchased primarily through an open internet marketplace continues without significant scientific testing. The Smart Tactical Application Tourniquet (STAT, STAT Medical Devices, LLC, Freehold, New Jersey) first appeared for sale in 2017 on first responder supply websites. While social media and a direct sales website (www.statmeddevices.com) advertise ease of use among laypersons for effective hemorrhage control, there is scant published research. The company readily advertises that the STAT’s non-elastic plastic material and zip-tie-like ratcheting strap "can stop bleeding within 5-10 seconds." This 76.2-cm ratcheting strap likely provides an intuitive layperson application with little training with written directions for placement on the device itself and simplicity with a self-locking capability, which may confer ease of use and lend itself to inexperienced users in stressful trauma situations. However, direct testing of the STAT with the CAT is limited. Only three out of 24 people were successful in applying the STAT to their own thighs, according to a Crisis Medicine Group online test (www.crisis-medicine.com). Success was defined as the absence of a posterior tibialis pulse. A small pilot study of 13 layperson volunteers found significantly lower median arterial occlusion pressures with the STAT compared to the CAT (216 mm Hg vs. 354 mm Hg), as well as lower rates of successful application (20% vs. 67%) [18]. To date, no other comparative evidence exists evaluating arterial occlusion efficacy or documented application success.

We sought to expand the study by directly comparing the application of the CAT and STAT devices for hemorrhage control efficacy when applied by laypersons after a brief standardized video instruction.

## Materials and methods

Ethics

The US Army Regional Health Command - Central Regulatory Office reviewed protocol C.2019.038e and determined it was exempt from institutional review board review. Consent was obtained, with a waiver of consent documentation granted.

Subjects and settings

All aspects of this study were conducted at Brooke Army Medical Center (BAMC), a level 1 trauma center caring for active duty military, veteran, and civilian patients located in San Antonio, Texas. Participation was anonymous. All non-military individuals over the age of 18 without declared medical professional experience were invited to participate through solicitation at the main entrance to BAMC. Medical professional experience was defined as any formal professional medical training or professional credentialing training, including emergency medical technician, paramedic, medical school, nursing, military medicine, or other formal collegiate or technical vocation training. Additionally, potential volunteers were excluded if they were unable to get in a position to apply the tourniquet (i.e., kneeling position) or had a medical condition that would preclude participation.

**Figure 1:**
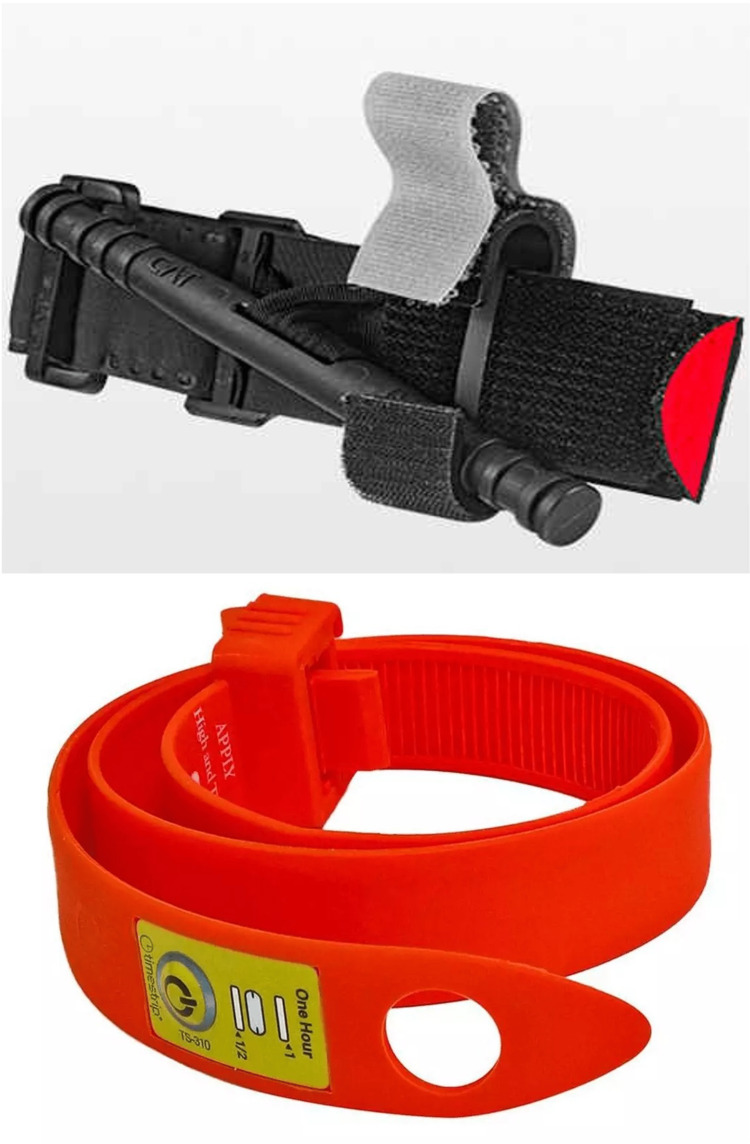
Comparison of the CAT (top) and the STAT (bottom) used in testing.

After screening and completing a brief demographic survey, volunteers were randomized to a device group using an online research randomizer tool (www.randomizer.org). Demographic questions included whether laypersons had completed any prior tourniquet application training (to include a formal Stop the Bleed Course) or had previous real-world experience in the application of a tourniquet device and were randomized to apply either the CAT generation 7 or STAT device (Figure 1). A new tourniquet device was used for each iteration of testing.

Volunteers then viewed a brief standardized instructional video on the proper application of their respective device created by the tourniquet manufacturer [19,20]. These videos were shortened and standardized to 60 seconds to only show instructions of device application and eliminate any promotion of individual device benefits so as to reduce bias, identical to previously published literature [18].

**Video 1:** Combat application tourniquet instructional video provided to volunteers.

**Video 2:** Smart tactical application tourniquet instructional video provided to volunteers.

Investigation team members oriented volunteers to the tourniquet application model and then read volunteers a standard short scenario description of an injured victim on the ground after an explosion in a public location. Volunteers were instructed to apply the tourniquet provided to the injured victim to stop the bleeding from the wound.

**Figure 2:**
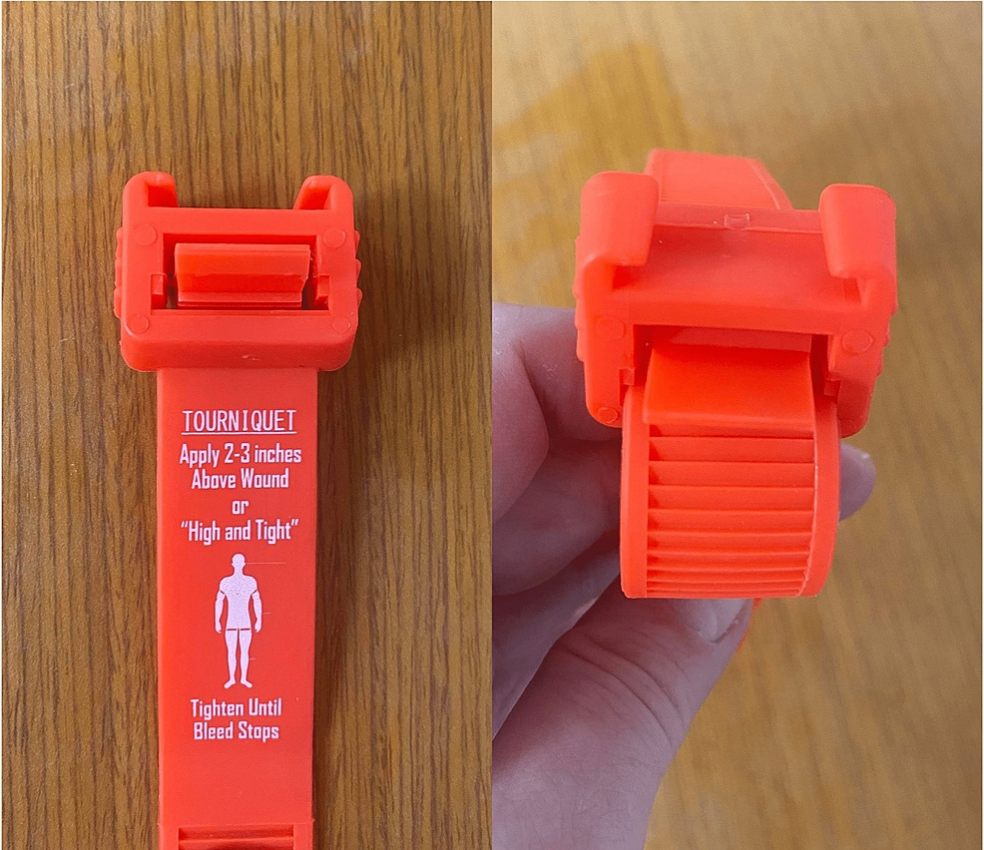
STAT printed instructions (left) and zip tie-like ratcheting mechanism (right).

Although not alerted to the instructions on the side of the STAT device, this section was deliberately not covered so as not to limit the potential benefits of printed instructions (Figure 2).

Simulation model

**Figure 3:**
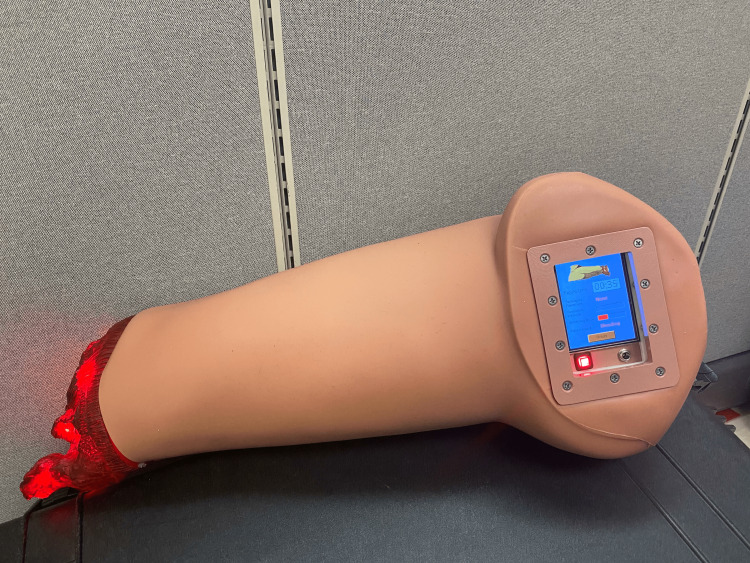
The HapMed manikin trainer used in testing.

A HapMed medical manikin trainer (CHI Systems, Plymouth Meeting, PA) simulated a victim with a lower extremity hemorrhage. The HapMed contains sensors that provide real-time feedback on the arterial occlusion pressure (measured in millimeters of mercury - mm Hg) of the tourniquet applied, the estimated blood loss volume in milliliters (mL), patient status (dead, bleeding, stable), and time to stop bleeding (Figure 3).

Volunteers were informed that the trainer has red flashing LED lights at the distal end of the limb to simulate bleeding. The flashing sequentially slows with increasing arterial occlusive pressure, eventually stopping altogether with complete hemorrhage control. They were able to visualize this LED light feedback mechanism throughout their participation. This was the only feedback volunteers received during their tourniquet application. The display with simulator feedback metrics was only visible to the study team members. Of note, this is the same trainer model that is utilized in the STAT advertised instructional video, where with a timed application of four seconds, an occlusion pressure of 518 mm Hg is stated to be achieved with a blood loss volume of 35 mL [20].

As with our prior research, this study utilized a standardized scenario of a "medium" body size patient with an initial bleeding rate of 625 mL per minute and a bleed-out time of 240 seconds. Trial time started when the volunteer verbally indicated they were starting, the volunteer grabbed the tourniquet, or the volunteer touched the manikin leg. Trial termination occurred when volunteers verbalized the perceived success of the tourniquet application, manikin patient status was read as "dead" (after 2500 mL total blood loss), or volunteers otherwise terminated the scenario. Upon scenario completion, volunteers completed a questionnaire for device feedback.

Our primary outcome was successful hemorrhage control, defined as a patient status of "stable" on the HapMed manikin trainer monitor at the completion of the scenario, and correct placement of the tourniquet on the extremity as determined by the manikin feedback and investigation team assessment (Table 1).

**Table 1:** Study metrics assessed. mL: milliliters; mm Hg: millimeters of mercury.

Metric	Measured by	Unit of outcome	Statistical analysis
Success (yes/no)	HapMed Manikin Trainer, investigating team member	Patient status “stable” on trainer monitor, and correct tourniquet placement on the extremity as determined by the manikin feedback and investigation team	Chi-square test
Occlusion pressure	HapMed Manikin Trainer	mm Hg	t-test
Blood loss volume	HapMed Manikin Trainer	mL	t-test
Instructional video helpfulness	User feedback	Likert scale	Mann-Whitney U test
Comfort applying tourniquet in real-world scenario	User feedback	Likert scale	Mann-Whitney U test

In accordance with CoTCCC recommendations, adequate tourniquet placement involved placing the distal end of the device at least 2 inches (5 centimeters) proximal to the injury [21]. Secondary outcomes included time to occlusion (in seconds, if applicable), time to scenario termination (in seconds), occlusion pressure achieved (in mm Hg), total blood loss (in mL), and volunteer feedback for ease of use and comfort on a 5-point Likert scale.

In consultation with a statistician, a power analysis assessing a 30% difference in tourniquet application with an alpha of 0.05 and powered to 80% required 84 total subjects. To select a population sample representative of the general population of interest, subjects were enrolled into four cohorts to normally match for age and gender variances: (1) females less than 55 years old; (2) males less than 55 years old; (3) females aged 55 years or older; and (4) males aged 55 years or older. Based on the 2014 census data, half of the population in the city of San Antonio is 18-54 years old, and 20% is 55 years old or older. Maintaining that proportion and using a sample size of 42 subjects per tourniquet group, 30 subjects were aged 18-54 years, and 12 subjects were aged 55-85 years.

We performed all analyses using Microsoft Excel (version 10, Redmond, Washington) and JMP Statistical Discovery from SAS (version 15, Cary, North Carolina). We analyzed the primary outcome proportion of tourniquet application success using a Chi-square test. Secondary outcomes of continuous variables such as blood loss and tourniquet pressure were analyzed with a t-test. Mean 5-point Likert items were tested for distribution and then assessed using the Mann-Whitney U test.

## Results

A total of 84 volunteers completed the study over a five-week period, with 42 applying the CAT and 42 applying the STAT. No volunteers reported any prior tourniquet application training or real-world application. For the primary outcome of successful hemorrhage control with tourniquet application, we found a statistically significant difference in favor of the CAT (21 of 42 applications vs. 0 of 42 applications, p < 0.001; Table 2).

**Table 2:** Tourniquet application rates of successful hemorrhage control.

	Successful % (n)	Unsuccessful % (n)	p-value	Total
CAT	50.0 (21)	50.0 (21)	<0.001	42
STAT	0.0 (0)	100.0 (42)		42

Volunteers applied the CAT with significantly higher average occlusion pressure (409.9 mm Hg, standard deviation [SD] 106.4 mm Hg) than those in the STAT cohort (116.5 mm Hg, SD 109.2, p < 0.001; Table 3).

**Table 3:** Tourniquet pressure and blood loss volume. mL: milliliter; mm Hg: millimeters of mercury; SD: standard deviation.

	Occlusion pressure (mm Hg)		Blood loss volume (mL)	
	Mean (SD)	p-value	Mean (SD)	p-value
CAT	409.9 (106.4)	<0.001	577.8 (309.5)	<0.001
STAT	116.5 (109.2)		974.6 (311.9)	

Similarly, the CAT application demonstrated significantly less blood volume loss (577.8 mL, SD 309.5) compared to the STAT (974.6 mL, SD 311.9, p < 0.001). Of note, 24 of 42 applications with the STAT (57.1%) demonstrated pressures less than 100 mm Hg, including 10 of 42 applications (23.8%) with an inability to register any pressure with the application.

Volunteers found each video segment to be helpful based on a 5-point Likert scale, although the average feedback was significantly higher for the CAT (4.7 SD 0.5 vs. 4.0 SD 1.0, p < 0.001, Table 4).

**Table 4:** User feedback. SD: standard deviation.

	Instructional video helpfulness		Comfort applying tourniquet in real-world scenario	
	Mean (SD)	p-value	Mean (SD)	p-value
CAT	4.7 (0.5)	<0.001	4.0 (0.8)	<0.001
STAT	4.0 (1.0)		2.4 (1.5)	

Volunteers also demonstrated a significantly greater level of comfort on average with the CAT over the STAT (4.0 SD 0.8 vs. 2.4 SD 1.5, p < 0.001).

## Discussion

In this fully powered prospective evaluation, we compared the application success rates of CAT and STAT by non-medical laypersons on a simulated lower extremity model. Our findings demonstrate significantly higher rates of hemorrhage control in CAT applications, and most notably, there were no STAT applications with successful hemorrhage control. Additionally, the CAT demonstrated significantly greater occlusion pressure, less blood volume loss, and better user feedback, all favoring the CAT. These findings support those found in the previous limited pilot study [18].

Tourniquets are, by definition, devices employed to halt the cessation of arterial blood flow [22,23]. Suboptimal tourniquet pressures create vascular issues aside from the basic lack of arterial occlusion. Lower tourniquet pressure may occlude venous return despite ongoing arterial flow, exacerbating blood loss and increasing the complication risk of hypovolemic shock, compartment syndrome, and death [24]. Suboptimal occlusion pressures demonstrated by the STAT in this sample population could potentiate these complications. While the previous pilot study found a median occlusion pressure of 216 mm Hg with the STAT, pressures registered as low as 113 mm Hg, which is slightly higher than the average pressure demonstrated in this population [18]. This could be in part due to different demographics within a sample population approximately nine times larger than the pilot study, including a greater diversity of age. In contrast, prior literature has determined that the CAT can achieve greater than 200 mm Hg of occlusion pressure even before engagement of its windlass [22].

Lower occlusion pressures found with the application of the STAT may stem from its pull-through design, which lacks a leverage mechanism. This method resembles the strap-and-buckle concept utilized by the Vietnam-era military standard-issue tourniquet that proved largely ineffective [25]. Failure to leverage this strap beyond initial pulling pressure, either by windlass or other means, may hinder the STAT from attaining sufficient occlusion pressures.

Additionally, tourniquet width demonstrates an inverse relationship with the pressure necessary for arterial occlusion [12,24]. Not all modern tourniquets use the same width, instead employing other mechanical means to attain optimal pressure. At 2.54 cm wide, the STAT is approximately 30% narrower than the CAT Generation 7 and does not reach the minimum 3.81 cm width deemed a critical requirement to be considered by the CoTCCC [12]. In addition to the lacking mechanism of application described above, the STAT’s width may further jeopardize its ability to consistently provide arterial occlusion.

When combined with online demonstrations showing 87.5% failure in the STAT as well as significantly worse performance in the pilot study, the poor performance trends compared to the CAT are troubling. With continued professional and public support of first responder and layperson programs such as the Stop the Bleed campaign, research on new hemorrhage control devices must likewise continue for objective comparison and the development of best practices. Considering these findings, the efficacy of the STAT is of continued concern.

Volunteers endorsed significantly higher levels of comfort with the CAT application compared to the STAT based on a 5-point Likert scale as part of a post-participation survey. This may be due to performance self-perception when seeing the LED light feedback from the HapMed medical manikin trainer, which could indicate hemorrhage control success or failure. Future studies should seek to further elucidate these findings with further surveys.

Our study is primarily limited by a convenience sample at a single institution. While volunteers generally found the video instruction helpful and the videos were standardized to 60-second clips from the manufacturers themselves, the videos may not be optimal for brief tourniquet instruction. Manufacturer videos may be more educational in their entirety; however, the investigating team sought to ensure both were of equal length to standardize instruction. It is noted that the CAT instructional video directs viewers to apply the tourniquet within two inches of the injury, while the STAT manufacturer’s video does not; however, this was believed to be mitigated by placement instructions printed on the side of the STAT device directing application "2-3 inches above the wound or ‘High and Tight’." The tourniquets were tested on a controlled model simulation and not on actual patients, thereby limiting the expansion of the findings to real-life hemorrhage scenarios. The HapMed manikin trainer has received criticism for its reliability and replicability of real-life hemorrhage scenarios, including the lightbulb feedback that may not ideally replicate hemostasis patterns with hemorrhage control; however, we hope to mitigate this issue through its standardized use across all volunteer applications [26]. The distance of the tourniquet from the wound site and the standardized settings on the HapMed manikin trainer could likewise influence results and could be expanded in the future to other settings to further validate these results. Finally, we relied on genuine volunteer participation in both their efforts of tourniquet application and accuracy in their responses on the surveys provided and therefore assumed no perception bias.

## Conclusions

In this randomized observational study, the CAT demonstrates significantly greater rates of success, arterial occlusion pressures, and end-user feedback, with additional decreased blood volume loss, compared to the STAT in the application by non-medical laypersons on a lower limb hemorrhage model. Our findings inform a growing body of literature on suboptimal STAT efficacy, with continued emphasis on larger and more robust studies assessing these devices. Future studies may expand upon these findings to include the use of in vivo studies; however, at the present time, concerns continue to exist regarding STAT efficacy for extremity hemorrhage.
